# Boosting the Activation of Molecular Oxygen and the Degradation of Rhodamine B in Polar-Functional-Group-Modified g-C_3_N_4_

**DOI:** 10.3390/molecules29163836

**Published:** 2024-08-13

**Authors:** Jing Chen, Minghua Yang, Hongjiao Zhang, Yuxin Chen, Yujie Ji, Ruohan Yu, Zhenguo Liu

**Affiliations:** 1 Key Laboratory of Flexible Electronics of Zhejiang Province, Ningbo Institute of Northwestern Polytechnical University, Ningbo 315103, China; 2 Department of Chemical and Material Engineering, Quzhou College of Technology, Quzhou 324002, China; yangmh@qzct.edu.cn (M.Y.); zhanghongjiao@qzct.edu.cn (H.Z.); 3 Department of Chemistry, Lishui University, 1 Xueyuan Road, Lishui 323000, China; chenyuxin@lsu.edu.cn (Y.C.); jiyujie@lsu.edu.cn (Y.J.); yuruohan@lsu.edu.cn (R.Y.); 4 School of Flexible Electronics and Henan Institute of Flexible Electronics, Henan University, Zhengzhou 450046, China

**Keywords:** polar functional group, g-C_3_N_4_, degradation, visible-light photocatalysis

## Abstract

Molecular oxygen activation often suffers from high energy consumption and low efficiency. Developing eco-friendly and effective photocatalysts remains a key challenge for advancing green molecular oxygen activation. Herein, graphitic carbon nitride (g-C_3_N_4_) with abundant hydroxyl groups (HCN) was synthesized to investigate the relationship between these polar groups and molecular oxygen activation. The advantage of the hydroxyl group modification of g-C_3_N_4_ included narrower interlayer distances, a larger specific surface area and improved hydrophilicity. Various photoelectronic measurements revealed that the introduced hydroxyl groups reduced the charge transfer resistance of HCN, resulting in accelerated charge separation and migration kinetics. Therefore, the optimal HCN-90 showed the highest activity for Rhodamine B photodegradation with a reaction time of 30 min and an apparent rate constant of 0.125 min^−1^, surpassing most other g-C_3_N_4_ composites. This enhanced activity was attributed to the adjusted band structure achieved through polar functional group modification. The modification of polar functional groups could alter the energy band structure of photocatalysts, narrow band gap, enhance visible-light absorption, and improve photogenerated carrier separation efficiency. This work highlights the significant potential of polar functional groups in tuning the structure of g-C_3_N_4_ to enhance efficient molecular oxygen activation.

## 1. Introduction

Synthetic dyes are common organic pollutants found in wastewater from industries, such as cosmetics, textiles, paints, and leather processing. Rhodamine B (RhB) is a water-soluble organic dye known for its industrial advantages, including its low cost and high color fastness [1]. Unfortunately, RhB exhibits potential neurotoxicity, genotoxicity, and carcinogenicity. Even trace amounts (approximately 1.0 mg/L) of RhB can have significant colorimetric effects on water, posing threats to both the environment and human health [2]. Several traditional methods have been developed to remove RhB from water, such as adsorption, membrane filtration, degradation, and coagulation [3]. Advanced oxidation processes (AOPs) are effective methods for removing difficult-to-degrade and non-biodegradable compounds, which makes them promising for RhB removal. Various advanced oxidation processes have been studied for RhB degradation, including ozone oxidation [4], Fenton and Fenton-like reactions [5,6,7], and photocatalysts [8,9]. These processes focus on generating reactive oxygen species (ROS) for degrading pollutants.

Molecular oxygen, as an environmentally friendly oxidant, is widely used in the field of environmental remediation [10]. By converting into reactive oxygen species (ROS), such as superoxide (·O_2_^−^), hydrogen peroxide (H_2_O_2_), hydroxyl radical (·OH), and singlet oxygen (^1^O_2_), molecular oxygen can exhibit excellent performances in pollutant degradation and clean energy conversion [11]. Therefore, promoting the activation of molecular oxygen into ROS has garnered significant research interest in recent years. Nevertheless, the direct oxidation of molecular oxygen is limited due to spin-forbidden reactions [12]. Traditional physical and chemical methods for activating molecular oxygen tend to have high energy consumption and low efficiency. This is mainly attributed to the high energy of the O=O bond (494 kJ/mol) [13]. Interestingly, photocatalysis presents a viable approach for molecular oxygen activation. Photogenerated electrons in the excited state can overcome the spin-forbidden barrier, transforming molecular oxygen into ROS [14]. Thus, developing high-performance photocatalysts through rational design emerges as a promising strategy to enhance activation efficiency [15,16].

Due to its abundant active edge sites and hierarchical nanostructures [17], g-C_3_N_4_ has emerged as a highly effective visible light-driven catalyst. It exhibits versatile applications in many fields, such as pollutant degradation [18], hydrogen evolution [19], CO_2_ reduction [20], and organic matter conversion [21]. However, the slow kinetic processes and the rapid recombination rate of photo-generated charges have significantly limited the application efficacy. Various modification strategies including doping, texture engineering, and semiconductor coupling have been pioneered [22,23]. Notably, functional group modification is a molecular doping process that optimizes the intrinsic conjugation system, optoelectronic properties, and energy band structure of g-C_3_N_4_. Novel functional groups, including hydroxyl, amino, carboxyl, cyano, urea, and carbon rings, have been continuously introduced to g-C_3_N_4_ to improve its photocatalytic performance [24].

Polar functional groups may induce local charge redistribution when introduced to the g-C_3_N_4_ matrix and surface. This results in effective local spatial charge separation, interfacial charge transfer, and a significant increase in carrier densities [25]. Li et al. developed surface-alkalized g-C_3_N_4_ (CN-KCl/NH_4_Cl), achieving a 3.2-times boost in photocatalytic H_2_ evolution. The addition of hydroxyl groups increased the conduction band potential, resulting in more reducing electrons and accelerating the rate of photoexcited electrons transferred to reactants [26]. Yu et al. enhanced the generation rate of H_2_ 11-fold through the surface hydroxylation of g-C_3_N_4_ using post-hydrothermal and plasmonic treatment [27]. Furthermore, polar functional group modifications can improve the interaction between the catalyst and the target. Nan et al. demonstrated that grafting hydroxyl groups to g-C_3_N_4_ might increase the affinity of the catalyst surface, allowing it to adsorb more CO_2_ and H_2_O molecules and effectively enhancing the photocatalytic process [28]. Polar functional groups can also improve the hydrophilicity of g-C_3_N_4_, facilitating a more effective dispersion of the catalyst in water and ensuring its proper utilization. Special procedures for preparing hydroxylation oxygen plasma [29,30], chemical oxidization [31], and hydrothermalization [32,33,34] have also been attempted. Nevertheless, the current processes would probably introduce unnecessary recombination centers for electron-hole pairs, which may cause a decrease in photocatalytic efficiency and chemical pollution. Furthermore, the complexity and high expense of the operation processes and unpredictable risks in the action process hinder the further development of these technologies. Therefore, a simple and environmentally friendly method to modify g-C_3_N_4_ with hydroxyl groups is highly desired.

Herein, we developed a novel hydroxyl-group-modified g-C_3_N_4_ (HCN) with ultrasonic-assisted hydrogen peroxide (H_2_O_2_). The introduction of hydroxyl groups into g-C_3_N_4_ promoted the interaction between photo-excited electrons and protons in water and enhanced the proton exchange process. The band gap energy of optimal samples was reduced, and the separation efficiency of photoexcited electrons and holes improved, resulting in a more favorable environment for the generation of reactive oxygen species. A significant improvement was observed in the photocatalytic performance of HCN. Specifically, the photocatalytic degradation constants of RhB for HCN-90 were 3.5 times higher than those for g-C_3_N_4_. This improvement was primarily attributed to the hydroxyl groups, which facilitated the mass transfer and interfacial charge transfer processes.

## 2. Results and Discussion

### 2.1. Characterization of g-C_3_N_4_ and HCN

**Figure 1:**
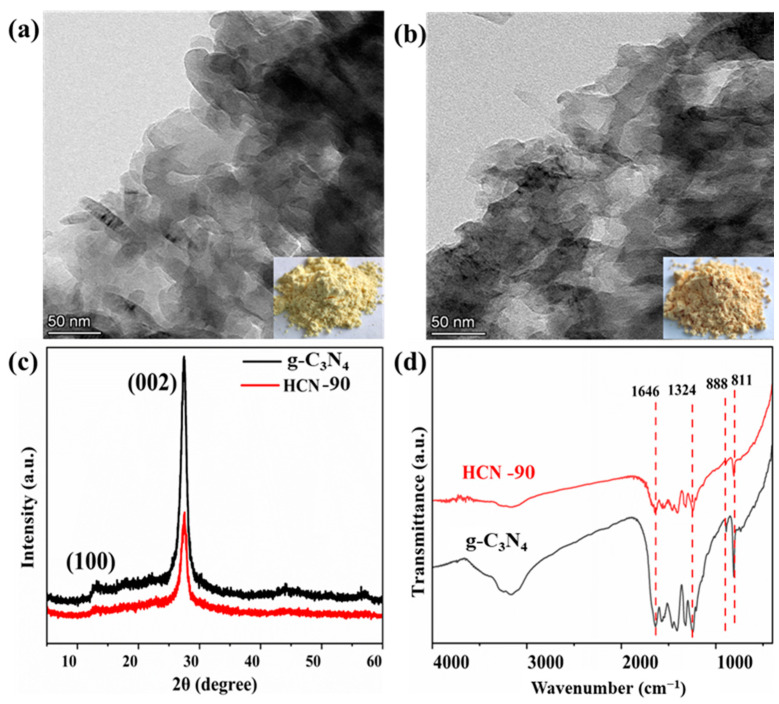
(**a**) TEM images of g-C_3_N_4_; (**b**)TEM images of HCN-90; (**c**) XRD patterns of g-C_3_N_4_ and HCN-90; (**d**) FT-IR spectra of g-C_3_N_4_ and HCN-90.

TEM images of the g-C_3_N_4_ and HCN-90 are shown in Figure 1a,b, respectively. Notably, HCN-90 and g-C_3_N_4_ exhibited similar morphology. HCN-90 exhibited a darker color to g-C_3_N_4_. This difference could be attributed to the presence of more polar functional groups on the surface of HCN-90, altering its electronic arrangement and the conjugation system. Next, XRD analysis was performed to investigate changes in crystal phase structures and crystallinity (Figure 1c). Both g-C_3_N_4_ and HCN-90 showed two characteristic diffraction peaks of g-C_3_N_4_ (JCPDS No. 87-1526) at 2θ = 27.5° (002) and 13.0° (100) [35]. These peaks corresponded to the tri-s-triazine ring in-plane compression and interlayer stacking structure of the conjugated aromatic system, respectively [36]. The significant decrease in the (001) diffraction peak of HCN-90 indicated disruption in the orderly stacking structure within the tri-s-triazine planes. Compared to g-C_3_N_4_, the diffraction peak at (002) for HCN-90 showed a slight rightward shift from 27.5° to 27.6°, indicating a reduction in the interlayer spacing [37]. Furthermore, the intensity of the (002) plane in HCN-90 decreased, supporting the conclusion of a shortened interlayer distance. Narrower interlayer distances were beneficial for faster charge transfer [38].

FT-IR spectroscopy was employed to assess the structure of g-C_3_N_4_ and HCN-90 (Figure 1d). HCN-90 exhibited a significant increase in broadband intensity in the 3000–3500 cm^−1^ region compared to g-C_3_N_4_. These broad peaks could be attributed to N-H and O-H stretching vibrational modes [39]. The increased broadband strength in HCN-90 indicated the formation of abundant surface hydroxyl groups. In the region of 1200–1650 cm^−1^, these bands corresponded to the typical stretching mode of C-N heterocycles [40]. Specifically, the peaks at 1324 cm^−1^ reflected the out-of-plane bending vibration characteristic of the heptazine ring [41]. The absorption band at 888 cm^−1^ was attributed to an N-H deformation mode [42], indicating an incomplete condensation of the amino groups. The sharp absorption peak around 811 cm^−1^ was associated with the typical breathing mode of the tri-s-triazine unit [43]. A similar structure indicated that the structure of HCN-90 remained unchanged.

**Figure 2:**
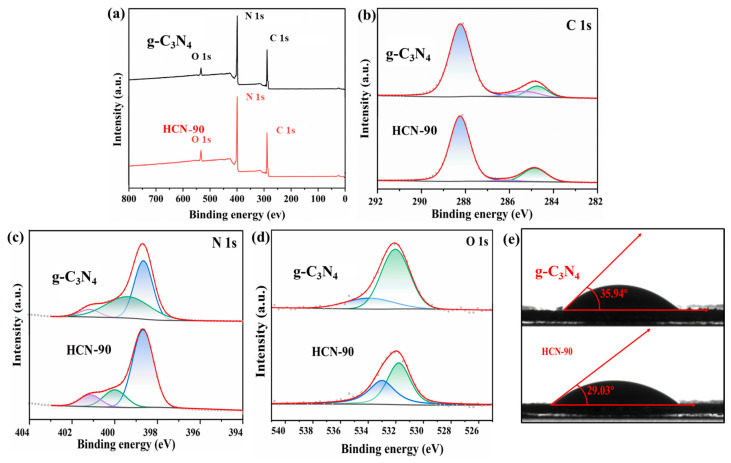
XPS spectra of g-C_3_N_4_ and HCN-90: (**a**) survey; (**b**) C 1s; (**c**) N 1s; (**d**) O 1s; (**e**) water contact angles.

XPS analysis was conducted to examine the surface composition and chemical states of g-C_3_N_4_ and HCN-90. In the XPS survey spectra (Figure 2a), O 1s, N 1s, and C 1s peaks were observed. Elemental analysis further revealed that the O-atom contents of g-C_3_N_4_ and HCN-90 were 3.06% and 6.36%. These results suggested the introduction of oxygen-containing functional groups in the samples following treatment with H_2_O_2_. The high-resolution C 1s spectrum (Figure 2b) of g-C_3_N_4_ exhibited three distinct peaks. The peak at 284.8 eV corresponded to the indeterminate carbon (sp3 C-C), whereas those at 286.3 eV and 288.7 eV arose from the C-N and N=C-N in the heptazine heterocycles [44]. The high-resolution N 1s spectra (Figure 2c) displayed peaks at 398.5 eV, 400.1 eV, and 401.0 eV, corresponding to C-N=C, C-N(-C)-C, and C-NH_2_, respectively [45]. The high-resolution O 1s spectra (Figure 2d) revealed two O species in g-C_3_N_4_ and HCN-90, with binding energies of 531.3 eV (O-H) and 533.8 eV (O-N). The former corresponded to surface hydroxyl groups, while the latter originated from intermediate products of melamine thermal polymerization [46]. Notably, the calculated atomic percentages of the O-H of g-C_3_N_4_ and HCN-90 were 19.81% and 47.51%, respectively. This suggested that the oxygen species predominantly existed as hydroxyl groups with hydroxyl on the surface. The contact angle (CA) measurements further supported the results, as shown for HCN-90 and g-C_3_N_4_ (Figure 2e). The reduced CA could be attributed to the abundance of hydroxyl groups on the surface.

**Figure 3:**
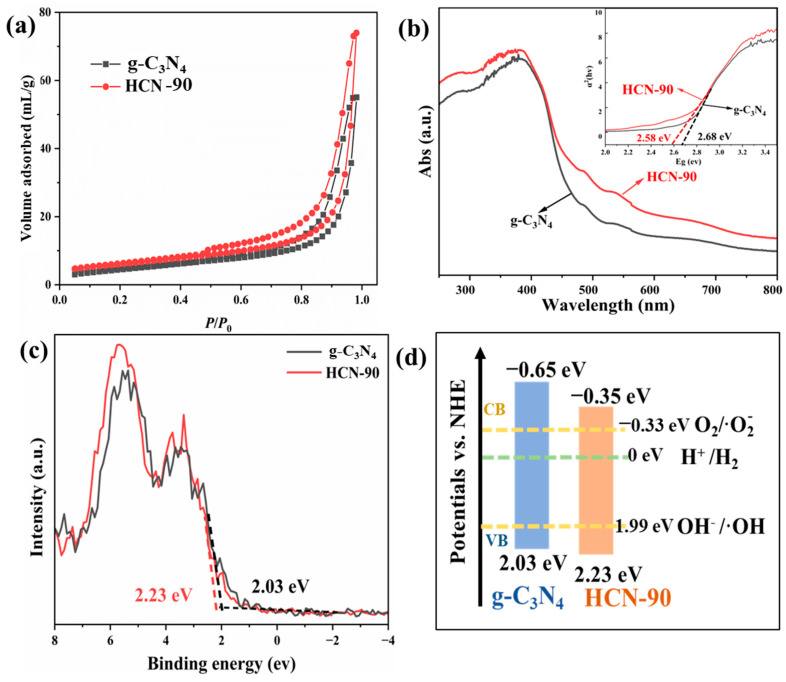
(**a**) Nitrogen adsorption–desorption isotherms; (**b**) UV–vis diffuse reflectance spectra; (**c**) XPS valence band spectra; (**d**) band alignments of g-C_3_N_4_ and HCN-90.

Figure 3a shows the N_2_ isothermal adsorption–desorption curves of g-C_3_N_4_ and HCN-90. Both curves exhibited typical IV adsorption patterns with H3-type hysteresis loops, indicating the presence of mesopores and macropores in both samples. The smaller hysteresis loop observed in g-C_3_N_4_ at high *P*/*P*_0_ was ascribed to the aggregation of g-C_3_N_4_ particles. Importantly, the specific surface area of HCN-90 measured 27.3 m^2^/g, surpassing the 11.5 m^2^/g recorded for g-C_3_N_4_. This expanded the specific surface area and provided numerous active sites for adsorption and surface reactions, effectively enhancing the photocatalytic reaction.

The photo-redox reaction is primarily governed by the potentials of valence bands (VBs) and conduction bands (CBs), as well as the bandgap energy of materials. Consequently, further investigation into the band structure and electronic conductivity was conducted. Diffuse reflectance spectroscopy (DRS) was used to characterize the light absorption properties. As shown in Figure 3b, the absorption edge of HCN-90 showed a red shift compared to that of g-C_3_N_4_. The red shift in the absorption edge correlated with a change in color from yellow in g-C_3_N_4_ to dark yellow in HCN-90. According to the Kubelka–Munk equation, the band gap of HCN-90 and g-C_3_N_4_ could be deduced [9]. The band gap of g-C_3_N_4_ was 2.70 eV, consistent with previously reported results [8]. For HCN-90, the value was approximately 2.58 eV.

The valence band X-ray photoelectron spectroscopy (VB XPS) spectrum in Figure 3c was used to estimate the band edge potentials of g-C_3_N_4_ and HCN-90. The VB potentials for g-C_3_N_4_ and HCN-90 were measured at 2.03 eV and 2.23 eV, respectively. The positive shift in the VB of HCN-90 enhanced the oxidizing capacity of holes and improved its photo-oxidation capability. In combination with the results of the DRS, the CB edge potentials of g-C_3_N_4_ and HCN-90 were estimated at −0.65 eV and −0.35 eV, respectively. The reduction in the CB facilitated the transfer of surface charge carriers and promoted the generation of reactive oxygen species.

Figure 3d illustrates the comprehensive influences of the polar functional group modifications on the band structure, including the potentials of key reactions. The potential of the CB remained more negative than the redox potentials of O_2_/·O_2_^−^, suggesting that photogenerated electrons could potentially be trapped by adsorbed O_2_. This trapping mechanism resulted in the production of reactive species, preventing light-induced carrier recombination [47]. The suitable band structure thermodynamically enabled boost-photo-induced charge separation and molecular oxygen activation.

### 2.2. Photoelectric Property

**Figure 4:**
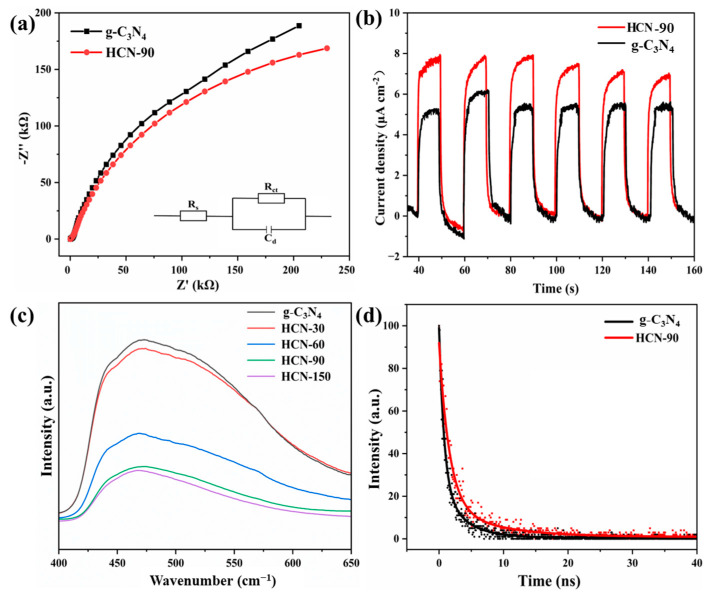
(**a**) EIS Nyquist plots; (**b**) transient state photocurrent spectra; (**c**) steady-state fluorescence spectra; (**d**) time-resolved fluorescence spectra.

Energy transfer for the activation of molecular oxygen was greatly facilitated by improving intersystem crossover processes to reduce non-radiative attenuation [48]. Electrochemical impedance spectroscopy (EIS) was employed to study the interfacial charge transfer capability. Figure 4a displays the EIS Nyquist plots of g-C_3_N_4_ and HCN-90, with the inset showing the corresponding equivalent circuit model for analysis. In the equivalent circuit model, *R*_ct_, *C*_d_ and *R*_s_ represented charge transfer resistance, double layer capacitance, and the electrolyte solution resistance, respectively. The smaller arc radius observed for HCN-90 compared to g-C_3_N_4_ indicated lower surface impedance and faster charge transfer in HCN-90. This observation supported the proposition that polar functional groups contribute to the enhanced separation of photogenerated electrons and holes, thereby improving photocatalytic activity [49].

The transient photocurrent response of the samples was measured and is shown in Figure 4b. All samples exhibited a rapid and reproducible photocurrent response during each illumination cycle. Notably, the photocurrent of HCN-90 was observed to be larger than that of g-C_3_N_4_, confirming the constructive role of polar functional groups in enhancing charge migration and separation. Fluorescence spectroscopy provided valuable insights into the separation and recombination of photogenerated electron-hole pairs. In Figure 4c, the photoluminescence intensity of g-C_3_N_4_ was stronger than that in HCN, and the fluorescence intensity of HCN gradually decreased with prolonged H_2_O_2_ treatment time. This phenomenon was likely attributed to the abundant polar functional groups, which enhanced charge trapping and effective charge transfer [50]. Consequently, this prolonged the charge carrier lifetime and contributed to the improvement in photocatalytic activity. The fluorescence decay curves were employed to explore the transfer efficiency of carriers. As illustrated in Figure 4d, the findings were fitted using the tri-exponential decay function. The average lifetime increased from 2.51 ns (g-C_3_N_4_) to 4.78 ns (HCN-90). The extended charge carrier lifetime of HCN-90 could be attributed to the electrical interaction between hydroxyl groups and graphitic carbonitride. These findings indicated that HCN-90 has better electron-hole pair separation.

### 2.3. Visible-Light Photocatalytic Activity Measurements

**Figure 5:**
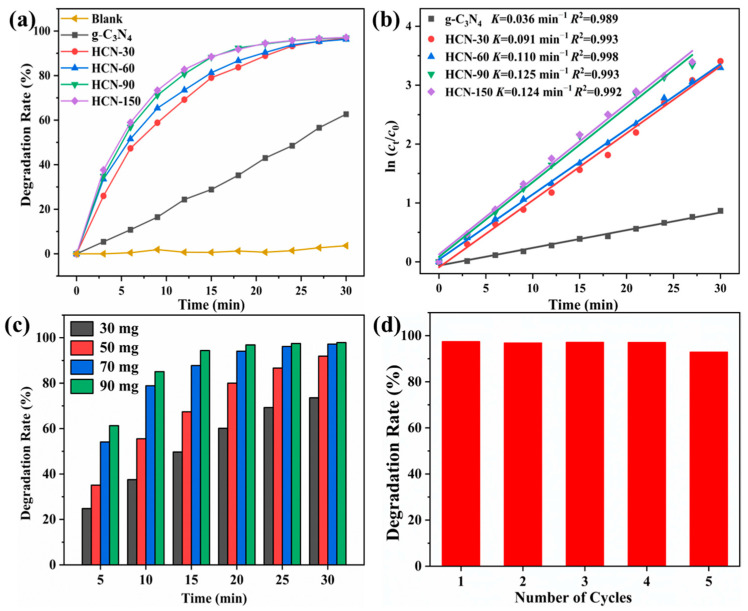
(**a**) Photocatalytic degradation of RhB; (**b**) first-order kinetics plot; (**c**) effect of dosage on degradation rate; (**d**) reusability of HCN-90.

The photocatalytic activities were assessed by monitoring the photodegradation of RhB in visible light irradiation. As shown in Figure 5a, the degradation rate showed no significant changes without the photocatalyst, indicating that the RhB degradation was caused by photodegradation rather than self-decomposition. The degradation rate of pure g-C_3_N_4_ was only 59.4%, whereas HCN consistently achieved degradation rates above 96%. The photocatalytic activities of HCN gradually increased with ultrasonic-assisted hydrogen peroxide treatment time. The results demonstrated that modification with hydroxyl groups significantly improved the photocatalytic activity, likely due to enhanced separation of photogenerated carriers and a narrowed band gap, thereby boosting the activation of molecular oxygen.

The kinetics of the photocatalytic degradation of RhB were further investigated. A first-order linear relationship was observed in the plots of ln (*c*_t_/*c*_0_) versus irradiation time (Figure 5b). The calculated reaction rate constants (*K*) were 0.036, 0.091, 0.110, 0.125, and 0.124 min^−1^ for g-C_3_N_4_, HCN-30, HCN-60, HCN-90, and HCN-150, respectively. Apparently, HCN-90 demonstrated the highest kinetic parameters, approximately 3.5 times that of g-C_3_N_4_. The enhanced photocatalytic performance of HCN-90 could be attributed to its larger specific surface area and improved charge carrier separation. The higher specific surface area increased chemisorption and mass transfer. The enhanced charge separation improved the photo responsiveness, contributing to the overall improved catalytic activity. Notably, HCN-90 was selected for further studies due to cost and time constraints, even though HCN-150 showed comparable photocatalytic performance.

Further investigation was conducted into the usage conditions of HCN-90. Figure 5c depicted the effect of HCN-90 dosage on the photodegradation of RhB. With increased HCN-90 dosage, the degradation rate of RhB also increased. At dosages of 30 mg, 50 mg, 70 mg, and 90 mg, the degradation rates of RhB within 30 min were 72.9%, 92.07%, 96.9%, and 97.7%, respectively. Higher catalyst dosages resulted in increased active substances, thereby enhancing the photodegradation rate within a specific range. However, excessive photocatalysts could only lead to the increased adsorption of pollutants. Moreover, a high concentration of suspension in the solution might hinder incident light penetration, thereby reducing photodegradation efficiency [51]. Ultimately, the optimal dosage of HCN-90 was found to be 70 mg.

Additionally, the reusability of HCN-90 was evaluated through five consecutive reaction cycles (Figure 5d). Over the five cycles, the photodegradation rates of RhB by HCN-90 were 98.8%, 97.4%, 96.8%, 97.0%, and 92.8% within 30 min, respectively. This indicated that HCN-90 exhibited negligible deactivation, underscoring its commendable cycle stability.

**Figure 6:**
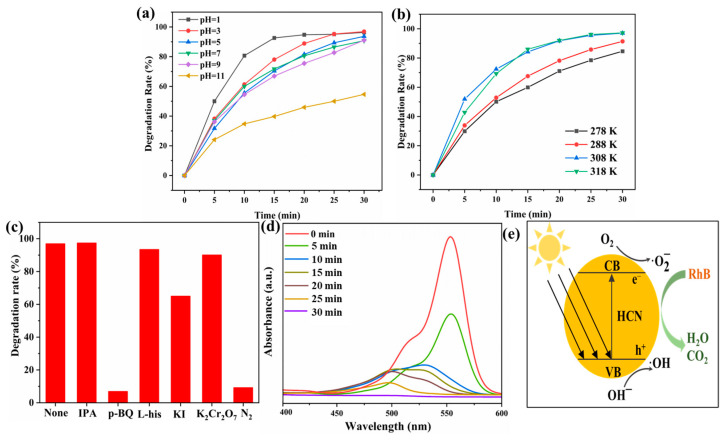
(**a**) Effect of pH on degradation rate; (**b**) effect of temperature on degradation rate; (**c**) effect of scavengers on degradation rate; (**d**) absorption spectra of RhB during the photocatalytic process by HCN-90; (**e**) possible mechanism schematic for RhB degradation under visible light irradiation.

The photocatalytic performance was strongly influenced by the solvent pH (Figure 6a). HCN-90 exhibited an optimal photocatalytic efficiency at pH = 1, achieving 99% degradation within 30 min. While there was a slight decrease in performance with an increasing pH within the range of 3–9, degradation rates remained above 85% within the same timeframe. However, at pH = 11, the degradation efficiency significantly declined to 55% after 30 min. This decline could be attributed to the pH-dependent structural characteristics of RhB, where protonation of the carboxyl group occurred when the solution pH was below the pKa of RhB (3.70) [51,52]. Figure 6b illustrates the effect of temperature on the photocatalytic rate of HCN-90. At 278 K, 288 K, 308 K, and 318 K, RhB degradation rates were 84.6%, 92.1%, 97.0%, and 97.2% (within 30 min), respectively. The increase in the catalytic rate was mainly due to the accelerated molecular thermal motion, facilitating the activation process of pollutants on the catalyst surface.

To elucidate the photocatalytic mechanism, experiments were conducted using various radical scavengers to degrade RhB. As shown in Figure 6c, photocatalytic activities remained unaffected after adding IPA, L-his, and K_2_Cr_2_O_7_ as quenchers for ·OH, ^1^O_2_, and e^−^, respectively. The results suggested that ·OH, ^1^O_2_, and e^−^ were not responsible for the degradation of RhB. However, introducing p-BQ and KI resulted in the RhB degradation rate decreasing from 96.9% to 6.9% and 64.9%, respectively. These findings indicated that ·$O_{2}^{-}$ radicals were the primary active component and h^+^ was also involved in the photodegradation of RhB. Furthermore, replacing O_2_ with N_2_ hindered the photocatalytic reaction, suggesting that O_2_ was essential.

The absorption spectra of RhB degradation with HCN-90 under visible light were shown in Figure 6d. Upon visible light irradiation, the characteristic peak of RhB at 554 nm gradually decreased and exhibited a blue-shift. Over the first 10 min, the peaks shifted from 552 nm to 497 nm, indicating the decomposition of RhB into N,N,N′-triethyl-rhodamine (539 nm); N,N′-diethyl-rhodamine (528 nm); N-ethyl-rhodamine (502 nm); and Rhodamine (497 nm) [53]. In the final 5 min, the maximum peak declined without an obvious peak, suggesting the further degradation of RhB into small molecules such as CO_2_ and H_2_O [54]. Thus, RhB photodegradation involved de-ethylation and the destruction of the conjugated structure [52]. RhB degradation was further confirmed by LC–MS, and the results are shown in Figure S1. Based on the mass spectra and molecular weight analysis, the catalysis process led to the degradation of RhB.

Based on these results, a proposed mechanism for the enhanced photoactivity of HCN is illustrated in Figure 6e. HCN was excited by photon energy, generating electrons and holes to form electron-hole pairs. The electron acceptor adsorbed on the surface of HCN induced photogenerated electrons in the CB and reacted with O_2_ to produce ·$O_{2}^{-}$. The ·$O_{2}^{-}$ exhibited strong reducibility and participated in the degradation of RhB. Additionally, holes in the VB could react with OH^−^ to produce highly oxidizing ·OH. However, due to the high potential of OH^−^/·OH, only a small amount of ·OH was produced in the photocatalytic process. Therefore, ·$O_{2}^{-}$ and h^+^ as the main active substances were responsible partially or completely for redox RhB to achieve a degradation effect. The comparison of this study with recently published results on the photocatalytic degradation of RhB is presented in Table 1. HCN-90 exhibited the efficient catalysis of RhB degradation under visible-light irradiation (420 nm), showcasing excellent reusability and rapid degradation rates. These findings demonstrated its practical applicability in wastewater remediation.

## 3. Materials and Methods

### 3.1. Materials

Melamine (C_3_H_6_N_6_, >99.0%), hydrogen peroxide (H_2_O_2_, ≥30%), p-benzoquinone (p-BQ, C_6_H_4_O_2_, >99%), potassium iodide (KI, ≥99%), and potassium dichromate (K_2_Cr_2_O_7_, ≥99.8%) were supplied by Adamas Chemical Reagent Co., Ltd., Shanghai, China. Rhodamine B (RhB, C_28_H_31_ClN_2_O_3_, >98.0%), isopropyl alcohol (IPA, C_3_H_8_O, >99.5%), and L-histidine (L-his, C_6_H_9_N_3_O_2_, >99.0%) were supplied by Tokyo Chemical Industry Co., Ltd., Tokyo, Japan.

### 3.2. Preparation of HCN

Pure g-C_3_N_4_ was synthesized via thermal polycondensation. In detail, 5 g of melamine was placed into an alumina crucible (Titan Technology Co., Shanghai, China) and heated in a muffle furnace (Yiheng Scientific Instrument Co., Shanghai, China) at 550 °C for 4 h, with a heating rate of 5 °C/min. Subsequently, the product was further heated at 550 °C for another 4 h to obtain the g-C_3_N_4_. HCN was synthesized through ultrasound-assisted oxidation with hydrogen peroxide. Typically, 0.5 g of g-C_3_N_4_ was mixed with a 7.5 mL H_2_O_2_ solution (30 wt%) and subjected to ultrasonicated using bath sonication (Dekang Cleaning Electronic Appliance Co., Shenzhen, China, power 60 W, frequency 40 kHz) for 30–150 min. Finally, the products were washed with deionized water three times and collected through centrifugation. The final products were obtained by drying at 60 °C overnight, while the corresponding solids were named HCN-x (x is the time of ultrasonication).

### 3.3. Characterization

X-ray diffraction (XRD) measurements were conducted by using a Bruker D8 Advance X-ray powder diffractometer (Bruker Co., Billerica, MA, USA) equipped with Cu Kα radiation (λ = 1.5406 Å) and a scanning speed of 10°/min. The accelerating voltage and emission current were set at 40 mV and 40 mA, respectively, with a scanning range from 5° to 60°. Transmission electron microscopy (TEM) imaging was performed using a JEOL-2010 transmission electron microscope (JEOL Ltd., Akishima, Japan). Fourier Transform Infrared Spectra (FT-IR) were collected with a Nicolet iS50 Spectrometer (Thermo Fisher Scientific Inc., Waltham, MA, USA) using KBr as diluents. The surface chemical state was analyzed by X-ray Photoelectron Spectroscopy (XPS) using a Thermo Fisher Escalab 250Xi apparatus (Thermo Fisher Scientific Inc., Waltham, MA, USA) with an Al-Kα source. The water contact angle was recorded by sessile drop analysis Dataphysics OCA20 (DataPhysics Instruments, Filderstadt, Germany). Specific surface areas were determined by Brunauer–Emmett–Teller (BET) analysis using a Micromeritics automatic surface area analyzer Gemini 2360 (Shimadzu Co., Kyoto, Japan) with nitrogen adsorption at 77 K. Ultraviolet-Visible Diffuse Reflectance Spectra (UV–vis DRS) were recorded with a Model Shimadzu UV 2550 spectrophotometer (Shimadzu Co., Kyoto, Japan) in the range of 250 nm to 800 nm. Time-resolved fluorescence spectra were obtained using an Edinburgh FLS 900 (Edinburgh Instruments Ltd., Edinburgh, England). A Waters SQD2 (Waters Co., Milford, CT, USA) Liquid Chromatography mass spectrometer (LC–MS) was used to identify the degradation intermediates of RhB. The mobile phase was methanol and a 0.2% methane acid solution. The gradient elution was programmed as follows: methanol was obtained at 70% and kept for 1 min, then linearly decreased to 10% over 8 min.

The electrochemical station employed was CHI 660D (Shanghai Chenhua Instrument Co., Shanghai, China), and the test system constituted a three-electrode system with a working electrode prepared by the drop-coating method, Ag/AgCl as the reference electrode, and Pt as the counter electrode. The electrolyte solution was 0.1 M Na_2_SO_4_. The working electrode was prepared by weighing 2.5 mg of catalyst and 50 μL of Nafion solution, followed by dispersion through ultrasonication with the addition of 0.45 mL of a water and ethanol mixture (1:9 volume ratio). A 20 μL drop of the dispersion was pipetted onto a square (1 × 1 cm^2^) of conductive glass and dried in a natural environment. Electrochemical impedance spectroscopy was performed in the frequency range of 0.05 Hz to 100 kHz at 0.7 V under irradiation.

### 3.4. Evaluation of Photocatalytic Activity

The photocatalytic degradation performances of samples were evaluated by using Rhodamine B (RhB) as the probe pollutant under visible light. Briefly, photocatalysts (70 mg) were weighed into an RhB solution (150 mL, 5 mg/L). After mixing, the reaction system was placed in the dark for 30 min to achieve adsorption equilibrium. Photodegradation was initiated by 420 nm LED (50 W) at room temperature, and the monitor wavelength was 554 nm. After the reaction, 5 mL of solvent was extracted from the reaction suspension to determine the RhB concentration. The degradation rate was calculated by using the following formula:

(1) $$\mathrm{Degradation}\;\mathrm{rate}\;(\%)=\frac{c_{0}-c_{t}}{c_{0}}\text{×}100\%$$

where *c*_0_ is the concentration of the reactant before illumination (mg/L), and *c*_t_ is the concentration of the reactant after a certain illumination period (mg/L). The initial pH (1, 3, 5, 7, 9, and 11), temperature (278 K, 288 K, 303 K, and 318 K), and dosage (30 mg, 50 mg, 70 mg, and 90 mg) on the degradation effect of Rh B (5 mg/L, 150 mL) were examined by changing the test conditions, respectively.

## 4. Conclusions

In summary, g-C_3_N_4_ modified with hydroxyl groups was prepared, characterized, and evaluated for its effectiveness in the photodegradation of RhB under visible light irradiation. By optimizing the duration of ultrasound-assisted oxidation with hydrogen peroxide, HCN-90 was synthesized, exhibiting a higher specific surface area, improved hydrophilia, and narrower interlayer distances. The hydroxyl groups on the surface acted as charge trapping centers, accelerating carrier separation efficiency. With a lower conduction band energy and narrower bandgap, HCN-90 facilitated molecular oxygen activation. Compared to pristine g-C_3_N_4_, HCN-90 demonstrated a 3.5-fold increase in the photodegradation rate of RhB (0.115 min^−1^). Even after five cycles, the photocatalytic efficacy of RhB degradation remained at 93%. Additionally, the RhB solutions were effectively degraded by HCN-90 under visible light irradiation within 30 min, resulting in a colorless solution. This bonding strategy presents a promising model for developing high-performance and eco-friendly photocatalysts for green molecular oxygen activation. The straightforward synthesis method, along with excellent recycling capabilities and rapid kinetics, positions HCN-90 as a viable candidate for industrial application.

## Figures and Tables

**Table 1:** The photocatalytic performance of different g-C_3_N_4_ composites for RhB photodegradation.

Catalyst	Irradiation Source	Irradiation Time (min)	Degradation Rate (%)	RhB Concentration (mg/L)	Catalyst Dosage (g/L)	Reusability	*K* (min^−1^)	Ref.
HCN-90	50 W LED	30	98.8	5	0.47	After five cycles: 93%	0.125	This work
g-C_3_N_4_/SmVO_4_	500 W Xe arc lamp	120	>90	10	1	After ten cycles: 93%	0.0345	[8]
TiO_2_@g-C_3_N_4_	300 W Xe arc lamp	100	93.3	4.79	1.33	After five cycles: 90%	0.021	[55]
40-OCN	30 W LED lamp	140	>90	30	1	After five cycles: >80%	0.0193	[56]
g-C_3_N_4_/CNTs	300 W xenon lamp	60	98.1	10	0.2	After five cycles: 90%	0.051	[53]
g-C_3_N_4_/AgI	500 W Xe lamp	100	73.86	20	0.67	After four cycles: >70%	0.0723	[57]
FeCN-7	300 W xenon lamp	60	-	20	2	After three cycles: 95%	0.117	[58]
CoFe_2_O_4_/g-C_3_N_4_	500 W Halogen lamp	120	57	-	0.6	After five cycles: 55%	-	[59]
CN-UM/Mt	300 W Xe lamp	60	almost 99	10	0.5	-	0.053	[60]
g-C_3_N_4_/5-rGO/SnO_2_	100 W halogen lamp	120	83.2	7.2	0.15	-	0.0285	[61]
Zn_2_Ti_3_O_8_/g-C_3_N_4_	400 W visible lamp	90	89.0	10	0.5	After five cycles: 83%	0.0211	[62]

## Data Availability

Data are contained within the article; further inquiries can be directed to the corresponding author.

## Supplementary Materials

The following supporting information can be downloaded at: https://www.mdpi.com/article/10.3390/molecules29163836/s1, Figure S1: (**a**) Total ion chromatogram and MS spectra of RhB intermediates at different retention times: (**b**) 0.99 min; (**c**) 0.48 min; (**d**) 0.32 min.
