# Supplementary material for: Boosting the Activation of Molecular Oxygen and the Degradation of Rhodamine B in Polar-Functional-Group-Modified g-C3N4
Source: Molecules. 2024 Aug 13;29(16):3836. doi: 10.3390/molecules29163836 (PMC11356892; doi:10.3390/molecules29163836)
Supplement: Supplementary file 1 [file molecules-29-03836-s001.zip › molecules-3125649-supplementary.pdf]

Supporting Information for:

Boosting the activation of molecular oxygen and the degradation of Rhodamine B over polar functional groups modified g-C_3N_4

Jing Chen [1,2,*], Minghua Yang [2], Hongjiao Zhang [2], Yuxin Chen [3], Yujie Ji [3], Ruohan Yu [3] and Zhenguo Liu [1,*]

[1] Key Laboratory of Flexible Electronics of Zhejiang Province, Ningbo Institute of Northwestern Polytechnical University, Ningbo 315103, China
[2] Department of Chemical and Material Engineering, Quzhou College of Technology, Quzhou 324002, Zhejiang, China
[3] Department of Chemistry, Lishui University,1 Xueyuan Road, Lishui 323000, China
* Correspondence: chenjing@qzct.edu.cn; iamzgliu@nwpu.edu.cn

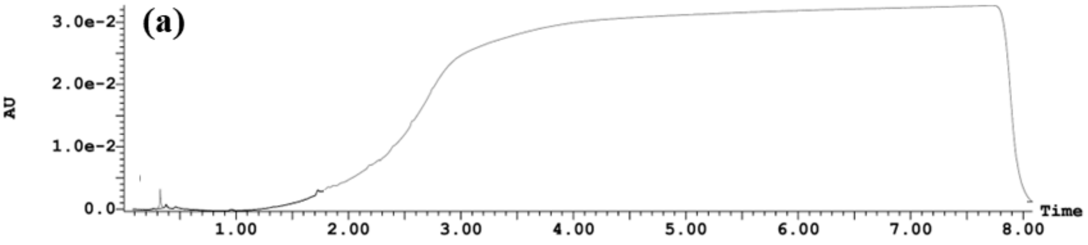

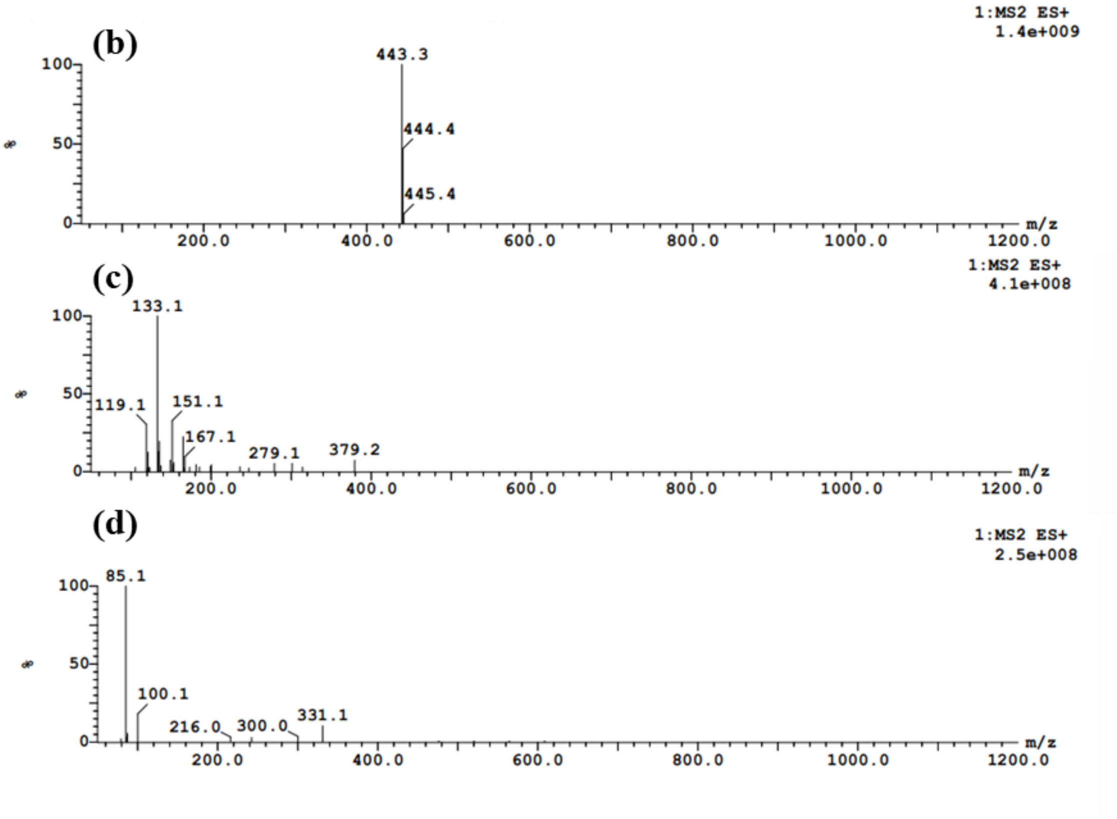

Figure S1. (a) Total ion chromatogram and MS spectra of RhB intermediates at different retention time (b) 0.99 min; (c) 0.48 min; (d) 0.32 min.
